# Torsion angular bin strings: algorithmic update and additional validation

**DOI:** 10.1186/s13321-026-01194-6

**Published:** 2026-04-13

**Authors:** Jessica Braun, Djahan Lamei, Philippe H. Hünenberger, Gregory A. Landrum, Sereina Riniker

**Affiliations:** Department of Chemistry and Applied Biosciences, ETH Zurich, Vladimir-Prelog-Weg 2, Zurich, 8093 Zurich Switzerland

**Keywords:** Torsional angle, Conformer space, Molecular shape

## Abstract

In our previous work, we introduced the concept of torsion angular bin strings (TABS), which is a discrete vector representation of a conformer’s torsional angles. Through this discretization, conformational states can be counted, yielding an estimate of the upper limit of the expected conformational ensemble size (nTABS). Besides nTABS being used as a quantitative measure of molecular flexibility, TABS itself is a way of grouping the conformers of a molecule without picking thresholds. This feature of TABS is especially valuable, as selecting suitable thresholds for metrics such as heavy-atom root-mean-square deviation (RMSD) or shape Tanimoto is highly system-dependent and can thus be challenging when working with large sets of molecules. Here, we describe the update to the nTABS algorithm of the TABS package since the last release. In addition, we present a classification study of conformer ensembles by TABS and compare it to classifications by a shape Tanimoto metric.

**Scientific contribution**

In contrast to our previous implementation, which handled molecular topological symmetry by enumerating all possible combinations that were simply permutations of one another, the new implementation treats TABS as mathematical objects governed by group theory, specifically Burnside’s Lemma. This approach requires substantially less code and delivers a notable improvement in computational speed. The study also builds upon our previously developed framework for categorization comparisons between TABS and heavy-atom RMSD. Here, we show the results of a similar comparison with a shape Tanimoto metric, which further support the hypothesis that TABS encode the shape of conformers in a meaningful way.

## Introduction

**Fig. 1:**
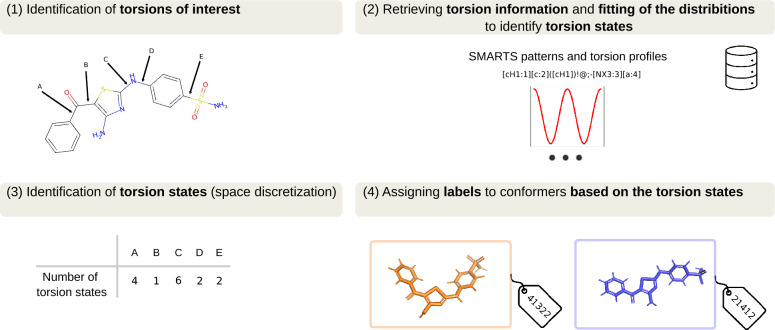
Standard TABS methodology [1] shown as an example TABS assignment for two example conformers of a molecule. Figure adapted from Ref. [1]. Licensed under CC-BY 4.0

The TABS methodology [1] is based on a predefined set of torsion patterns for rotatable bonds (where atoms involved in the bond have at least one non-hydrogen neighbor) [2, 3], for which the experimental torsional-angle preferences were determined based on the crystal structures in the Cambridge Structural Database (CSD) [4, 5]. For a given molecule, the applicable torsion profiles for each relevant dihedral are identified (Fig. 1). Through the *a priori* knowledge of the torsion profiles, the conformational space of a molecule can be discretized by isolating the peaks of the observed distributions. This discretization enables the assignment of unique labels to conformers that share the same state across all their dihedrals. Beyond providing an estimate of the size of the conformer space of a molecule (nTABS), the assignment of TABS labels enables rapid determination of whether two conformers are the same or different. A more in-depth explanation of the methodology is provided in Ref. [1].

## More efficient calculation of nTABS

**Fig. 2:**
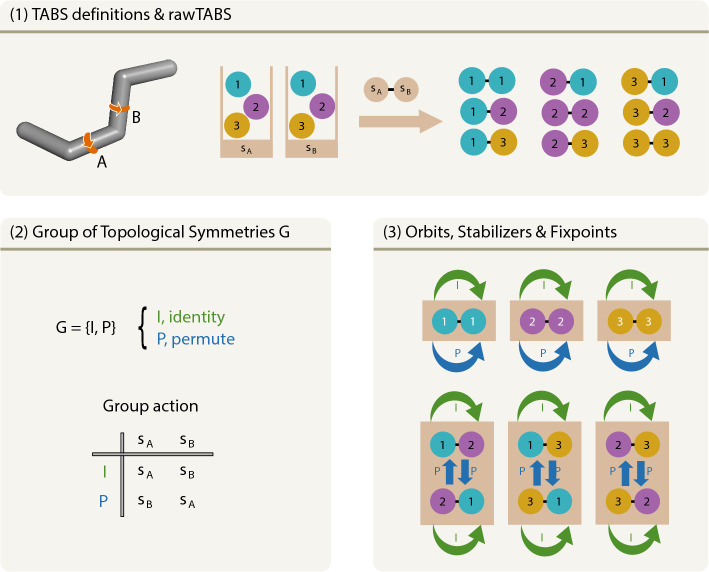
Illustration of the concepts needed for the intuitive explanation of Burnside’s Lemma in the context of TABS. (**1**) Description of all TABS quantities important for the nTABS calculation. Torsions of interest $N = 2$. Torsion states $s_A, s_B \in \{1,2,3\}$, hence multiplicities $m_A = m_B = 3$. The right hand side shows the set of rawTABS *S* with naive nTABS = $|S| = 9$. (**2**) The group of topological symmetries *G* to be applied on the set of rawTABS *S*. (**3**) Illustrations of the concepts of orbits, stabilizers, and fixpoints. All rawTABS are fixpoints under *I*. The fixpoints under *P* are marked in white

The conformational space accessible to a given molecule can be quantified by nTABS. In our previous work [1], nTABS for molecules with topological symmetry was calculated using exhaustive enumeration followed by filtering to remove redundant TABS, i.e., those describing the same torsional conformation based on a different atom numbering. However, the computational cost of this calculation grows exponentially with the number of torsional angles. Therefore, we introduce an alternative approach in this work. We use standard results from group theory to formulate a much more efficient algorithm for calculating nTABS. To make the mathematics more understandable, the concepts and quantities discussed below are illustrated in Fig. 2 considering the simple case of a molecule with two symmetry-equivalent torsional angles.

A TABS is a string of *N* integers, referred to as bits. Each bit *i* can take a value ranging from 1 to $m_i$, where $m_i$ is the multiplicity of bit *i*. We will refer to an arbitrary string of bits *s* with values within these ranges as a "raw" TABS (rawTABS), i.e., a TABS that has not been canonicalized. For a given number of bits and their associated multiplicities, the ensemble of all possible rawTABS forms a set *S*. Its cardinality (number of elements) is given by $M=|S|=\prod _{i=1}^N m_i$. Often, the graph describing the covalent structure of a molecule will present symmetries, referred to here as topological symmetries. Any permutation of the atom numbering that leaves the atom types and their connectivities unchanged is a topological symmetry. In the context of rawTABS, such a symmetry corresponds to a permutation of the bits, with all bit exchanges occurring at identical multiplicities. For a given molecular graph, the set of all such permutations (also including the identity) form a group *G*, its cardinality being |*G*|. The action of a symmetry $g \in G$ on a rawTABS $s \in S$ is noted as $g \cdot s$. Due to symmetries, different rawTABS may represent the same torsional conformation with different atom numberings.

For this reason, a TABS is not defined as a single rawTABS, but as an equivalence class of rawTABS, i.e., a set of rawTABS that can be interconverted via the topological symmetries of the molecule. In group theory, such a set is referred to as an orbit *Orb*(*s*). As explained in detail in Ref. [1], in the TABS methodology, the lexicographic smallest member is used as the canonical representation of the orbit.

For estimating the computational expense of the nTABS algorithm, two cases have to be distinguished:

1 $$\begin{aligned} \mathcal {O} = {\left\{ \begin{array}{ll} |G| = 1: & \mathcal {O}(N) \\ |G| > 1: & \mathcal {O}(|G|NM) \\ \end{array}\right. } \end{aligned}$$

In the following, we propose a computationally much more efficient alternative for the case $|G| > 1$.

Introducing the stabilizers *Stab*(*s*) defined as the set of all $g \in G$ which leave *s* invariant, the orbit-stabilizer theorem [6] states that

2 $$\begin{aligned} |Orb(s)|\ |Stab(s)| = |G|. \end{aligned}$$

This result accounts for the fact that the symmetries of *G* acting on *s* may produce either the same *s* (thereby extending the stabilizer set) or another *s* (thereby extending the orbit). Intuitively, this relationship implies that a molecule presenting a larger number of topological symmetries will possess a smaller number of distinct torsional conformers. Using this result, we could enumerate the rawTABS and then calculate |*Stab*(*s*)| instead of |*Orb*(*s*)|. The resulting algorithm would scale as $\mathcal {O}(|G|M)$, i.e., only slightly better than the previous approach. A serious gain is achieved when changing from an enumeration over rawTABS to an enumeration over symmetries.

For this purpose, we introduce the fixpoint set *Fix*(*g*) of symmetry *g* defined as the set of all rawTABS that are left invariant by *g*. Since the total number of invariances (i.e., of (*s*, *g*) for which $g \cdot s=s$) is independent of the summation order, one has

3 $$\begin{aligned} \sum _{s \in S} |Stab(s)| = \sum _{g \in G} |Fix(g)|. \end{aligned}$$

This allows us to rewrite $nTABS=|T|$ with $T = \{Orb(s)\}$ as

4 $$\begin{aligned} |T|\, = & \sum\limits_{{Orb \in T}} 1 = \sum\limits_{{Orb \in T}} {\sum\limits_{{s \in Orb}} {\frac{1}{{|Orb(s)|}}} } \, = \frac{1}{{|G|}}\sum\limits_{{Orb \in T}} {\sum\limits_{{s \in Orb}} | } Stab(s)|\, \\ = & \frac{1}{{|G|}}\sum\limits_{{s \in S}} | Stab(s)|, \\ \end{aligned}$$

where we used Eq. 2 and 3 as well as the fact that |*Orb*(*s*)| has the same value for all members *s* of an orbit. It follows that

5 $$\begin{aligned} nTABS = |T| = \frac{1}{|G|} \sum _{g \in G} |Fix(g)|, \end{aligned}$$

a result known in group theory as Burnside’s lemma [7] (sometimes also called the Cauchy-Frobenius lemma).

Using this lemma, we can calculate nTABS by looping over the |*G*| symmetries and determining the associated number of fixpoints. The latter calculation involves identifying independent groups of permuting bits and multiplying the multiplicities of these blocks, a task that scales as $\mathcal {O} (N)$. It follows that the overall scaling of the new algorithm is in $\mathcal {O} (|G|N)$, thereby removing the exponential increase of the computational cost with *N*.

## Comparison of TABS with shape-based categorization

We validate the original concept of TABS further by testing the hypothesis that a TABS categorization of a pair of conformers should correlate with a significant shape overlap as the similarity in all their torsions and hence their overall 3D structure should be reflected in a good shape score. For this, we applied the same procedure as previously [1] to compare the classification of conformers with TABS versus atom-positional root-mean-square deviation (RMSD). In this classification framework, each pair of conformers of an ensemble are either labeled as the same or different depending on their RMSD or TABS labels, respectively. From the resulting confusion matrix, the expected trend of increasing RMSD thresholds for molecules of increasing flexibility was reproduced when optimizing for maximal values of positive predictive value (PPV) and negative predictive value (NPV) [1]. The two metrics are defined as follows:

6 $$\begin{aligned} PPV = \frac{TP}{TP+FP}, \ \ NPV = \frac{TN}{TN+FN} \end{aligned}$$

**Fig. 3:**
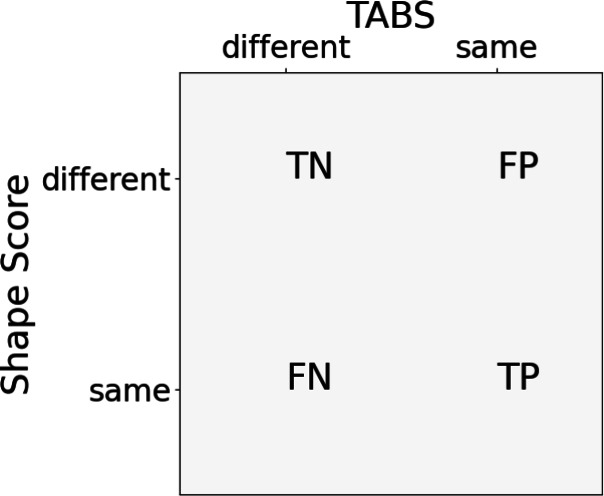
Schematic confusion matrix for the comparison between the TABS and the shape Tanimoto score. TN: true negatives, TP: true positives, FN: false negatives, FP: false positives

The PPV indicates how likely it is that a predicted positive is a true positive, while the NPV reflects the likelihood that a predicted negative is a true negative. Thus, if both metrics are equal to 1, there is complete agreement between the categorization metrics in classifying the data set. We hypothesized that this categorization framework should also result in reasonably high PPV and NPV values when comparing TABS with a shape Tanimoto similarity score (Fig. 3).

All shape-comparison calculations were performed on molecules from the Platinum data set [8]. Their grouping by their flexibility as described by nTABS [1] gives three subsets: low flexibility molecules (nTABS $< 500$), medium flexibility molecules (500 ≤ nTABS > 10,000), and high flexibility molecules (nTABS ≥ 10,000). The PubChem shape-based alignment code integrated within the RDKit [9, 10] was used to align the conformers and calculate the shape Tanimoto similarity scores with incorporated molecular features in form of the molecule’s canonical atom ranks. Using the canonical atom ranks as features forces the shape alignment to take the molecule’s underlying topology into account, allowing for a fair comparison between the shape Tanimoto and TABS categorization.

## Results

### Speed-up of the nTABS algorithm

In addition to the presented formal proof that calculating nTABS is an application of the Burnside’s lemma, the correctness of the implementation was also tested by calculating nTABS for the Platinum data set [8] and comparing the results to the previous values. For all 4548 molecules, the same nTABS value was obtained. The new implementation of the nTABS algorithm is not only more elegant, with a significant reduction in number of lines of code, but also comes with a substantial speed-up. For the entire data set, the naive implementation was timed at 108 s, whereas the new implementation only took 3 s, a 37-fold increase in speed (measured on Intel(R) Xeon(R) W-1270P CPU @ 3.80GHz).

### Comparing categorization with TABS versus shape tanimoto similarity

**Fig. 4:**
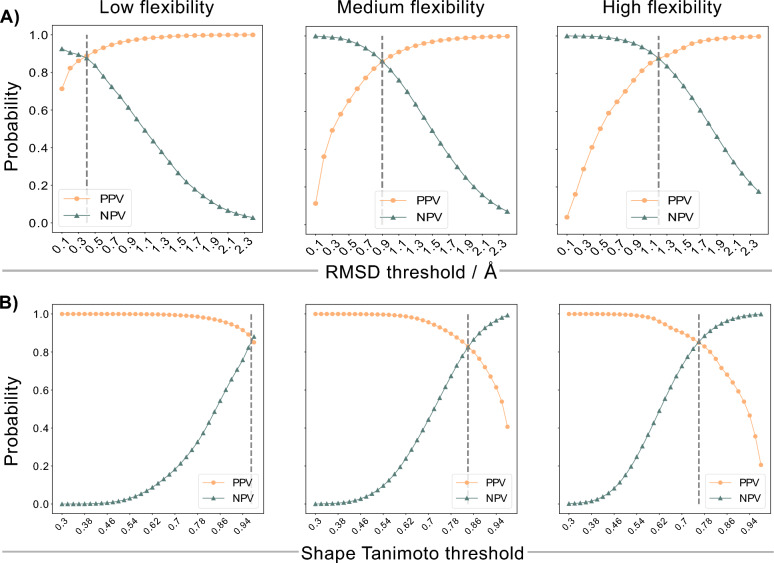
Positive predictive values (PPV, orange) and negative predictive values (NPV, dark green) as a function of a metric threshold (A: RMSD, B: shape Tanimoto) sorted into the different flexibility categories according to nTABS. **A**: Figure from Braun et al. [1], published by the American Chemical Society and licensed under CC-BY 4.0. The determined optimal RMSD thresholds in the three different flexibility categories are 0.4, 0.9 and $1.2~{\AA }$. **B**: The determined optimal shape Tanimoto similarity threshold is 0.97, 0.84, and 0.76 in the respective categories

The quality of the TABS categorization was evaluated against shape-aligned pairs of conformers of the same ensemble scored by the shape Tanimoto similarity score (see section comparison of TABS with shape-based categorization). Figure 4B presents the PPV and NPV values as a function of the shape Tanimoto threshold for the three flexibility categories. The same analysis between TABS and RMSD performed in Ref. [1] is shown for comparison (Fig. 4A).

As previously seen for the RMSD, Fig. 4B shows a similar size/flexibility dependence of the shape Tanimoto with the threshold ranging from 0.97 in the low flexibility category to 0.84 in the medium, and 0.76 in the high flexibility category. This trend is expected: As molecular flexibility increases, volume and atom counts grow, so a fixed relative difference corresponds to a larger absolute change in the shape Tanimoto similarity. Moreover, across all three flexibility categories, differentiating between the same or different conformer pairs based on TABS and the shape Tanimoto similarity achieved NPV and PPV values of roughly 80 % or higher, indicating strong agreement and supporting the earlier hypothesis that TABS and the shape Tanimoto metric can reach good agreement as both encode a shape description.

## Conclusions

We showed that the previous implementation of nTABS for molecules with topological symmetry can be replaced with a description following Burnside’s lemma. Beyond providing the mathematically correct formulation of the underlying concept, it results in a significant speed-up. Furthermore, we showed that TABS encode a shape description that correlates strongly with classifications of conformers as same or different based on the shape Tanimoto metric. This paper accompanies a major version release of the TABS code package.

## Data Availability

All code used to perform this study is open source and available on GitHub: https://github.com/rinikerlab/TorsionAngularBinStrings.

## References

[1] Braun J, Katzberger P, Landrum GA, Riniker S (2024) Understanding and quantifying molecular flexibility: torsion angular bin strings. J Chem Inf Model 64:7917–7924 39390326 10.1021/acs.jcim.4c01513 PMC11523068

[2] Schärfer C, Schulz-Gasch T, Ehrlich H-C, Guba W, Rarey M, Stahl M (2013) Torsion angle preferences in druglike chemical space: a comprehensive guide. J Med Chem 56:2016–2028 23379567 10.1021/jm3016816

[3] Guba W, Meyder A, Rarey M, Hert J (2016) Torsion library reloaded: a new version of expert-derived SMARTS rules for assessing conformations of small molecules. J Chem Inf Model 56:1–5 26679290 10.1021/acs.jcim.5b00522

[4] Allen FH (2002) The Cambridge structural database: a quarter of a million crystal structures and rising. Struct Sci 58:380–388 10.1107/s0108768102003890 12037359

[5] Groom CR, Allen FH (2014) The Cambridge structural database in retrospect and prospect. Angew Chem Int Ed 53:662–671 10.1002/anie.201306438 24382699

[6] Carter NC (2009) Visual group theory, 1st edn. pp. 198–199. Mathematical Association of America, Washington, DC

[7] Burnside W (1911) Theory of groups of finite order, 2nd edn. Cambridge University Press, Cambridge

[8] Friedrich N-O, Meyder A, Bruyn Kops C, Sommer K, Flachsenberg F, Rarey M, Kirchmair J (2017) High-quality dataset of protein-bound ligand conformations and its application to benchmarking conformer ensemble generators. J Chem Inf Model 57:529–539 28206754 10.1021/acs.jcim.6b00613

[9] Thiessen P. pubchem-align3d. https://github.com/ncbi/pubchem-align3d. Accessed 1 Jan 2025

[10] RDKit: Open-source cheminformatics. https://www.rdkit.org. Accessed 1 Jan 2025.

